# Aggression, Aggression-Related Psychopathologies and Their Models

**DOI:** 10.3389/fnbeh.2022.936105

**Published:** 2022-07-04

**Authors:** József Haller

**Affiliations:** Department of Criminal Psychology, Faculty of Law Enforcement, University of Public Service, Budapest, Hungary

**Keywords:** aggression, psychopathologies, violence, laboratory models, strength, limitations

## Abstract

Neural mechanisms of aggression and violence are often studied in the laboratory by means of animal models. A multitude of such models were developed over the last decades, which, however, were rarely if ever compared systematically from a psychopathological perspective. By overviewing the main models, I show here that the classical ones exploited the natural tendency of animals to defend their territory, to fight for social rank, to defend themselves from imminent dangers and to defend their pups. All these forms of aggression are functional and adaptive; consequently, not necessarily appropriate for modeling non-natural states, e.g., aggression-related psychopathologies. A number of more psychopathology-oriented models were also developed over the last two decades, which were based on the etiological factors of aggression-related mental disorders. When animals were exposed to such factors, their aggressiveness suffered durable changes, which were deviant in the meaning that they broke the evolutionarily conserved rules that minimize the dangers associated with aggression. Changes in aggression were associated with a series of dysfunctions that affected other domains of functioning, like with aggression-related disorders where aggression is just one of the symptoms. The comparative overview of such models suggests that while the approach still suffers from a series of deficits, they hold the important potential of extending our knowledge on aggression control over the pathological domain of this behavior.

## Introduction

Why are people aggressive and how can this behavior be kept under control? These two questions appear crucial in a world where over 20 million Disability-Adjusted Life Years are lost each year due to interpersonal violence, which ranks it the 18th on the list of the World Health Organization, ahead of many serious diseases including various forms of cancer (World Health Organization [WHO], 2022). Moreover, in terms of deaths causes, violence is in an even more “prominent” position (World Health Organization [WHO], 2015). Solutions to the problem of aggression are currently sought by various disciplines from sociology to psychology and criminology (McGuire, 2008; Meier and Wilkowski, 2013; Glowacki and McDermott, 2022).

Biomedical research is among the major research avenues to address the questions formulated above, for two reasons. Firstly, the most dangerous form of aggression, i.e., criminal violence is quite frequently associated with psychiatric disorders. Although estimates vary, the share of mentally disordered violent offenders was as high 90-100% in some studies (Freedman and Hemenway, 2000; Gostisha et al., 2014). Secondly, criminal violence is associated with a series of neural dysfunctions ranging from prefrontal deficits to amygdala dysfunctions and deficient prefrontal cortex-amygdala communication etc. (Raine and Yang, 2006; New et al., 2007; Belfry and Kolla, 2021). While the utility of sociological, psychological, and criminological approaches is unquestionable, it is widely believed that understanding the biological control of violence may lead to the development of better pharmacological and other tools of control (e.g., deep brain stimulation, [Bibr B71][Bibr B95]).

For ethical, practical, and technical reasons, a good deal of the biomedical research on aggression is performed in laboratory animals. This type of research always resorted to models, mainly because the behavior is relatively rare, its timing is difficult to predict, and it is often embedded in contexts that make its targeted study difficult under natural conditions. A multitude of models have been developed, which were rarely if ever compared systematically in terms of their utility to the understanding of psychopathological aggression. Here I will comparatively overview classical models that “transferred” natural behaviors into the laboratory and novel models that aimed at mimicking psychopathological states. The major question asked will be how these models are able to bring us closer to the understanding of aggression and violence.

## Classical Approaches

### The Principles Standing Behind Classical Approaches

According to a wide-spread view, there is a unique mechanism of aggression control, that may be “hyperactive” in violent people, but which essentially is not altered (Covington et al., 2019; for further details see below). As such, violence may be understood by studying aggression.

To be more accurate, there are three assumptions common to the classical models:

(1) Behavioral control in simple laboratory setups mirrors that seen in complex natural environments.

(2) The subjects of such studies – mostly rodents – are similar to humans to a degree that allows generalizations. Specifically, it is assumed that the control of aggression has some general rules that are valid for many if not all mammals, including humans.

(3) Unwanted (e.g., psychopathological) forms of aggression are controlled like the aggression displayed in these models. An extreme example of this assumption was the use of findings obtained in cat hypothalamic stimulation studies to explain criminal violence (Siegel and Douard, 2011).

### The Main Classical Models of Aggression

The oldest laboratory model is hypothalamic aggression, which is elicited by the electrical stimulation of a subregion of the hypothalamus, e.g., the hypothalamic attack area (Hess, 1928; Kruk, 1991). This model is classical in the meaning that it was used for almost one hundred years. Yet the behaviors elicited by stimulation are markedly different from those observed in other classical models and are reminiscent of those observed in models of psychopathological aggression. Therefore, stimulation models will be presented in the next section.

The most frequently used model in aggression research is the resident-intruder paradigm (Thurmond, 1975; Koolhaas et al., 2013), which consists in placing an unfamiliar animal, the intruder, into the home-cage of another, the resident. This results in a fight within minutes, which is practically over within 10-20 min, a very convenient timing from an experimental point of view. In addition, the simplicity of the setup, i.e., a cage that can be placed virtually anywhere allows the convenient application of various research techniques from the simplest (e.g., video recording for a detailed behavioral study) to the most sophisticated ones (e.g., optogenetics) (Koolhaas et al., 2013; Oliveira et al., 2021). Animals are believed to show territorial aggression in this paradigm, and as such, it is also assumed that the home-cage is equivalent with the territory where the animal obtains food, finds protection from predators, has access to mating partners, and raises its offspring, and which – being a valuable resource – is readily defended by their “owners” (residents). Naturally, the home-cage is a very simplified version of natural territories, especially in social species like the rat where territories are defended by the colony rather than by individuals alone (Schweinfurth, 2020). Yet it is assumed that the defense of territories under natural conditions is sufficiently similar to home-cage behaviors to allow the formulation of generally valid hypotheses on territorial aggression and on aggression in general.

An aggressive behavior different from that observed in the resident-intruder paradigm is seen in the fear-induced aggression models, where fear is triggered by the exposure of rodents to a larger conspecific (Blanchard and Blanchard, 1989), a predator (Blanchard et al., 1990), or humans (Plyusnina and Oskina, 1997). Under such conditions, rodents attack the source of fear especially when there are no escape routes. The behavior observed in this model is called defensive aggression as opposed to offensive aggression that is expressed in the resident-intruder paradigm. The two types of aggression show marked differences in the behaviors displayed and their neural control (Blanchard and Blanchard, 1989).

The shock-induced model of aggression, which was rather popular in the 70’s and 80’s, may be considered a variant of fear-induced aggression with the specification that the fear-inducing agent is inanimate in this case. Its presence in the early literature justifies the description of the paradigm here, but the procedure was rarely employed lately (but see Mamiya et al., 2017). The paradigm consists in the administration of electric shocks through a grid floor to two rodents placed in a neutral cage where spontaneous aggression is usually sporadic (Powell et al., 1969). Immediately after the administration of shocks, rodents start fighting. To our personal experience, the behavior elicited by shocks is a combination of offensive threats and defensive postures with biting attacks performed rarely.

A yet different type of aggression is displayed by subjects in the maternal aggression paradigm, which is based on pup-defense displayed by mothers. These violently attack unfamiliar male intruders which may kill their pups, but rarely attack female intruders, who pose no or minimal danger (Gandelman, 1972). In contrast to aggression displayed by males, the attack of mothers is violent, targets vulnerable body parts of opponents (usually the head and face), and is not signaled socially by threats (Parmigiani et al., 1988). Although this behavioral profile resembles that seen in models of abnormal aggression (see below) the behavior is functional in mothers and serves inclusive fitness, i.e., the transmission of genes to the next generation.

The visible burrow system is one of the main experimental setups that allows the study of aggression in the context of complex social environments (Blanchard et al., 1988). In this and similar models, rodent colonies live together for long periods of time in large enclosures that include burrows, e.g., natural living spaces for rodents. The colonies may be composed of males only or may be mixed-sex, in which case pups and juveniles will soon become parts of the colony. The paradigm allows the study of complex social relationships, e.g., the formation and maintenance of social hierarchies and their temporal evolution. Such colonies were also used to study the interaction between group-living and the ontogenetic development of emotionality (Kaiser et al., 2007) and the social integration of animals submitted to models of abnormal aggression (Tulogdi et al., 2014). Although ecologically highly valid, this model requires considerable laboratory space, costly living enclosures for subjects, and is labor-intensive. Probably for this reason, more simple variants of the model were developed, where the enclosure was smaller, lacked burrows, and the length of cohabitation was reduced to a couple of weeks (Millard and Gentsch, 2006; Mikics et al., 2007).

The behavioral variables studied in all these models are the latency of the first bite, biting attack counts, threatening and defensive postures, behaviors indicative of dominance relationships (dominant and submissive postures), amiable interactions (e.g., social investigation) and several aggression-unrelated activities like exploration and resting. Aggressiveness is considered increased when bite latencies are reduced, bite counts and the duration of threatening and dominant postures are increased. Decreased times devoted to defense and submission may also indicate increased aggressiveness. Increased aggression is usually associated with decreased amiable interactions.

### Strength and Limitations

The main advantage of classical aggression models is their high ethological validity and robustness. Studying aggression by these models is relatively simple because the behavior is expressed spontaneously once incentives and opportunities are present or are created in a laboratory context. Importantly, a variety of aggression types are modeled, which have their human equivalents, and as such, findings may be generalizable to humans (Blanchard, 2017).

The strength of such models, however, are at the same time their limitations. All the aggressions observed in classical models are functional. The defense of a territory and fighting for social rank (in the resident-intruder test and colony models, respectively) ensure access to food, mating partners and other resources. Escaping fearful situations by counterattacks serves survival when no other escape is available, whereas the defense of pups serves inclusive fitness. This is in line with the biological approach, which conceptualizes aggression as a form of competition that consists in the delivery of harm for gaining access to resources and reproduction. Although wording may differ from author to author, the essence of the approach remained the same over the last century (Craig, 1917; Jalabert et al., 2018). This approach has several implications, which are highly important from the point of view of modeling aggression and violence in the laboratory. Firstly, it involves that aggression *per se* is a natural behavior, moreover, indispensable for survival and genetic fitness. Not surprisingly, it is present in all the species that are anatomically able to perform it from worms, cephalopods, insects, to vertebrates and mammals (Fitzpatrick and Wellington, 1983; Reinhard and Rowell, 2005; Huffard et al., 2010). As such, aggression is maintained evolutionarily because it is adaptive. Secondly, being an essential behavior, it is naturally innate. Individuals can perform it without prior learning as shown by studies in animals raised in total social isolation (Harlow, 1965). Although animals and humans may improve their fighting abilities by experience (e.g., during play-fighting), the basic motor patterns are innate.

For the society, however, psychopathological forms of aggression are more problematic than functional aggression, not lastly because they are persistent and are embedded in an array of dysfunctions called mental disorders or psychopathological states. In contrast to functional aggression, mental disorders are no natural states. According to the Diagnostic and Statistical Manual of Mental Disorders (DSM), they are diagnosed when a perception, feeling, or behavior is inappropriate to the situation, it lasts long or occurs frequently over a prolonged period and causes distress and/or impairments in important areas of functioning (American Psychiatric Association, 2013). Although aggression-related psychopathologies may occur early, they are not inherited, and their development can be assigned to certain causes, called etiological factors.

Classical models undoubtedly contributed greatly to the understanding aggression as a biological phenomenon. One can rather confidently assume that behaviors observed in simple laboratory environments are analogous to those displayed in nature and studies in a variety of species including lower animals show that the mechanisms governing aggression are rather universal. For instance, serotonin decreases aggressiveness in fish, amphibians, reptiles, birds, rats, humans, moreover, octopuses (Deckel, 1996; Clotfelter et al., 2007; Ten Eyck, 2008; Mosienko et al., 2012; Dennis et al., 2013; Edsinger and Dölen, 2018; da Cunha-Bang and Knudsen, 2021). At the same time, however, it is questionable whether an innate and functional behavior shaped by evolution can model behavioral alterations prompted by environmental insults, i.e., etiological factors. Among other reasons supporting such doubts are studies showing that the neural mechanisms underlying deviant animal aggression are quite different from those underlying natural forms (see below).

## Models of Psychopathological Aggression

In principle, abnormal aggression models offer the possibility of understanding not aggression as a natural behavior but unwanted aggression as a psychopathological phenomenon. From a broader perspective, the development of abnormal aggression models marked a shift from the biological to the biomedical approach, which, as such, may provide a new way to answer the questions asked in the first sentence of this study.

### The Principles of Developing Psychopathologic Aggression Models

**FIGURE 1:**
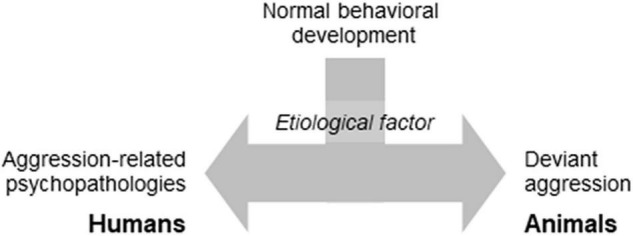
The basic idea standing behind the psychopathology-oriented aggression models.

Laboratory models of abnormal aggression assumed that humans and animals respond similarly to environmental insults. Consequently, the etiological factors of aggression-related psychopathologies would elicit behavioral changes that are analogous (to a certain extent at least) to those seen in disordered people (Figure 1). As such, the central element of an abnormal aggression model is the etiological factor, more accurately its laboratory model.

In the models developed so far, the effects of etiological factors on aggression were investigated in the resident-intruder test, one of the oldest models of aggression. In contrast to earlier practices, however, qualitative changes became more important than the classical quantitative measures (e.g., bite latency, bite counts etc.) because it was observed that under the effect of etiological factors the adaptive behavior transformed into a disruptive one (Haller et al., 2001; Haller and Kruk, 2006; Miczek et al., 2013).

Particularly, it was observed that rodents submitted to various etiological factors of aggression-related psychopathologies, the rules governing natural aggression were violated: aggression became unnecessarily dangerous, unpredictable to social partners, and unsafe for evolutionarily valuable individuals (females, pups). For the rules of evolutionarily sustainable aggression and their violation by animals exposed to etiological factors of aggression-related psychopathologies see Table 1. As such, the behavioral patterns observed in these models fulfills the basic criteria of psychopathologies set forth by the DSM-5 (American Psychiatric Association, 2013). Behaviors were inappropriate to the contexts where they were expressed, the condition was long-lasting; moreover, was difficult to correct, and caused significant impairments in critical areas of functioning. In addition, alterations in aggression were associated with a series of physiological dysfunctions, e.g., augmented, or blunted stress responses, and behavioral deficits, e.g., decreased sociability, increased anxiety-like and depression-like behaviors, deficits in social integration, etc. As with aggression-related psychiatric disorders, dysfunctional aggression seems to be embedded in a wider array of dysfunctions.

**TABLE 1:** Rules governing adaptive aggression and their violation in abnormal aggression models (i.e., in rodents submitted to the etiological factors of aggression-related psychopathologies).

Natural aggression	Abnormal aggression
Intent is signaled by threats to allow the opponent to avoid aggression.	Many or most bites are unpreceded by the social signaling of intention.
Intensity is limited to avoid exhaustion and to minimize the risk of injuries.	Bite counts are significantly larger than in controls and are delivered in persistent bursts.
Attacks are aimed at body parts that lack vital organs (e.g., the back and flanks in rodents) to avoid lethal injuries.	Attacks often target the head, throat, and belly, occasionally the paws and testicles. *
Submission and/or flight inhibits aggression, which permits withdrawal from the conflict.	Attacks are continued despite submission signals by the opponent
Reproductively valuable individuals (e.g., pups and females) are safe from aggression.	Females and pups are readily attacked under various (e.g., territorial) contexts.

The table was based on earlier work by Haller et al. (2001); Haller and Kruk (2006), Miczek et al. (2013), and Haller (2017). * The placement of bites is different in hamsters than in mice and rats. In juvenile hamsters, attacks are directed to the face and cheeks which may prematurely shift to adult-type attacks that are aimed at the belly and rump (Wommack and Delville, 2003). This shift in attack targeting may be considered an abnormal feature in this species.

### The Main Abnormal Aggression Models

There are a number of abnormal aggression models, each being based on a particular etiological factor of aggression-related psychopathologies. I will present these models below grouped according to the nature of etiological factors. For a summary, see Table 2.

**TABLE 2:** The main abnormal aggression models.

Etiological factor category	Model	References	Abnormal aggression symptoms
Genetics	Selection for aggression	Benus et al., 1991	D, E, F, P, V
	Selection for anxiety	Beiderbeck et al., 2012	E, F, V
Developmental stress	Early subjugation	Delville et al., 1998	A, E
	Peripubertal stress	Márquez et al., 2013	D, E, F, P, V
	Post-weaning social isolation	Tóth et al., 2008	D, E, P, V
Drugs	Alcohol	Miczek et al., 1992	E, V
	Anabolic androgenic steroids	Melloni and Ferris, 1996	E, F
	Cocaine	Ricci et al., 2005	E
Physiology	Hypothalamic stimulation	Hess, 1928	D, E, F, P, V
	Selection for stress responses	Walker et al., 2017	no data*
	Glucocorticoid deficit	Haller et al., 2001	D, P, V

Note that many models were developed long before the concept of abnormal animal aggression was developed. In their case, symptoms of abnormal aggression were identified considerably later, often by different authors. For instance, the SAL/LAL aggression selection lines were created in the early 90’s (Benus et al., 1991), yet the abnormality of the increased aggression of the SAL line was documented considerably later (Haller et al., 2006). A, accelerated development of aggression; D, disregarding social signals; E, excessive aggression; F, attacks on females; P, poor signaling of attack intentions; V, attacks on vulnerable targets; *, the paper reported on an “abnormality index”, without detailing this.

#### Genetics

The heritability of aggression-related psychopathologies is rather robust; it may account for over 50% of variance in aggression (attention deficit-hyperactivity disorder: 51%, Young et al., 2009; antisocial personality disorder: 51%, Rosenström et al., 2017; conduct disorder with callous-unemotional traits: 75%; Viding et al., 2013; psychopathy: 52%; Hicks et al., 2012). As such, heritability may be considered an etiological factor *per se*.

The heritability of aggressiveness prompted the development of selection lines for aggression. Three such selection lines were created so far: the short attack latency mice (van Oortmerssen and Bakker, 1981), the Turku aggressive (Lagerspetz and Lagerspetz, 1971), and the North Carolina 900 selection lines (Cairns et al., 1983). All three displayed excessive aggression; in addition, the short attack latency mice delivered attacks on vulnerable body targets (head, throat, belly), showed reduced signaling of attack intentions, disregarded appeasement signals (submission by the opponent), and attacked females (Haller et al., 2006; Natarajan and Caramaschi, 2010). As such, these selection lines displayed abnormal aggression, and may be used as models of aggression-related psychopathologies. In theory, they may be used to model the role of the genetic component of psychopathological aggression, especially so as the behavioral profile of the three lines is markedly different (Natarajan et al., 2009). This suggests that the selection process affected different sets of genes with different behavioral consequences, which may offer the opportunity to study the interaction between specific behavioral profiles and genetic backgrounds. This possibility was not exploited so far.

Symptoms of abnormal aggression – targeting bites on vulnerable body parts, deficient signaling of attack intentions and attacks on females – were observed also in rats selected for anxiety (Liebsch et al., 1998; Neumann et al., 2010; Beiderbeck et al., 2012). Remarkably, both the high-anxiety and the low-anxiety strain showed abnormal aggression, but their behavioral profile was different, which may be considered another unexploited opportunity to investigate the genetic underpinnings of specific and stable aggressive behavioral profiles.

#### Developmental Stress

It is believed that significant life events suffered in critical periods of life elicit persistent changes in behavior and may lead to the development of psychopathologies in general, and aggression-related psychopathologies in particular. Critical periods of life, when stressors lead to the delayed increase of the likelihood of aggression and aggression-related psychopathologies include the fetal period, when the effects of stressors are mediated by the mother (Salatino-Oliveira et al., 2016; Savolainen et al., 2018), early childhood (Wright et al., 2016; McLaughlin et al., 2017), and adolescence (Herrenkohl et al., 2001; Estrada-Martínez et al., 2011).

Fetal (maternal) and early (neonatal) stressors do change the behavior of adult rodents; yet the observed changes are consistent with increased anxiety and depression rather than aggression and in addition, data on abnormal aggression symptoms were conflicting (Sandi and Haller, 2015; Haller, 2020, p. 159). By contrast, stressors administered during adolescence resulted in the development of three important models of abnormal aggression.

The *early subjugation model* consists in repeatedly exposing hamsters and occasionally rats to defeats from adult males in their peripubertal period (postnatal days 28 to 42) (Delville et al., 1998; Cunningham and McGinnis, 2008). This early treatment durably increased aggressiveness in adulthood, accelerated the development of aggressive behavior such that adult forms of aggression were expressed in juveniles, and decreased the age-selectivity of aggression by promoting attacks on juveniles by adults (Wommack and Delville, 2003; Ferris et al., 2005). Subjugation stress also delayed sexual maturation and led to social avoidance (Bastida et al., 2009, 2014).

The *peripubertal stress model* is very similar to the early subjugation model except that the stressors administered to juveniles were non-social. Peripubertal rats were exposed successively at two-day intervals to the open field, fox odor, and an elevated platform, all which elicited stress responses. These ostensibly minor stressors dramatically increased the level of aggression in adulthood, elicited attacks on vulnerable body parts of opponents, decreased the signaling of attack intentions, and induced attacks on females and anaesthetized opponents (Cordero et al., 2012; Márquez et al., 2013; Papilloud et al., 2018; Walker et al., 2018). Interestingly, similar behavioral changes were observed in females exposed to the three stressors in the pubertal period (Cordero et al., 2013). Thus, the full spectrum of abnormal aggression symptoms was observed in the peripubertal stress model; moreover, changes were independent of gender.

The *post-weaning social isolation model* consists in rearing rats in individual cages from weaning to adulthood. When confronted with intruders as adults, rats submitted to this model showed high levels of aggression, a phenomenon observed long before the development of the concept of abnormal aggression (Potegal and Einon, 1989). Subsequent research showed that rats submitted to the model preferentially attacked vulnerable body parts of their opponents and did deficiently signal attack intentions by social threats (Tóth et al., 2008; Toth et al., 2011). In contrast to all the models presented above, aggression was expressed on the background of high behavioral agitation and exacerbated autonomic and glucocorticoid stress responses. Behavioral and physiological alterations were persistent and resistant to resocialization (Tulogdi et al., 2014). Notably, rats submitted to the model showed marked deficits in social integration when placed in social groups. A similar behavioral profile was observed in female rats submitted to the model (Oliveira et al., 2019). This study also showed that isolation rearing impaired social memory in both males and females and promoted attacks against juveniles in females.

Taken together, these findings show that stressors administered during puberty lastingly change behavior and favor the display of deviant forms of aggression in adulthood. Behavioral changes proved gender-independent in two of the three models, and aggression profiles – complemented with other physiological and behavioral deficits – were durable, moreover, resistant to simple interventions, e.g., resocialization.

#### Drugs

Human observations show that various drugs, among others alcohol, anabolic-androgenic steroids, and psychostimulants enhance aggressiveness (Bennett et al., 1970; Cherek et al., 1987; Licata et al., 1993; Su et al., 1993).

Such drugs may increase aggression shortly after their consumption and behavioral effects subside once drug effects fade away. Although temporary, such states are listed among mental disorders by the DSM-5 (American Psychiatric Association, 2013) under the term “intoxication”, e.g., “Alcohol intoxication” (DSM-5 p. 497) and “Stimulant intoxication” (DSM-5, p. 567). Both involve increases in anger and aggression, and aggression models were built for both. Alcohol self-administration consistently increased aggression in a subgroup of laboratory rodents, whereas in others aggression remained unchanged or even decreased (Miczek et al., 1992, 2013). This situation is highly similar to the human case, where the effects of alcohol on aggression also show high individual variation. For a long time, the arguments for the abnormality of alcohol-induced aggression were increased aggressiveness, and the emergence of lasting attack bursts that are uncharacteristic to the species studied (Miczek et al., 2013). It was, however, recently shown that alcohol intoxication changed attack targeting (Newman et al., 2018), which is one of the key symptoms of abnormal aggression. Amphetamine also increased aggression and in addition, it prompted attacks on females (Gobrogge et al., 2009), one of the signs that may render aggression abnormal.

In other cases, drugs may have long-lasting effects on behavior, especially when exposure covers sensitive life periods, e.g., adolescence. For instance, anabolic androgenic steroids administered during puberty to hamsters dramatically increased aggression in adulthood, and accelerated the development of adult aggression forms, which, as shown in Table 1 may be considered a sign of behavioral abnormality in this species (Melloni and Ferris, 1996; Morrison et al., 2014; Lee et al., 2021). The administration of anabolic androgenic steroids to adults has similar behavioral consequences (Ambar and Chiavegatto, 2009). Cocaine treatment during puberty also increases aggression in adulthood (Ricci et al., 2005). Unfortunately, no other measures of abnormal aggression were studied with this model.

Taken together, these findings show that (i) The administration of certain drugs increases aggression acutely, which may be considered a model of psychopathological aggression elicited by drug intoxication. (ii) Certain compounds have long-term effects on aggression when animals are treated during their puberty. Although symptoms of abnormal aggression were studied sporadically, the few available data suggest that beyond increased aggression levels, the aggression displayed in these models is deviant.

#### Physiological Models

In this section I present models that were based on physiological phenomena known to be associated with aggression-related psychopathologies rather than on their etiological factors. Particularly, I present the hypothalamic stimulation model, work in rats selected for high stress responses, and the glucocorticoid deficiency model of abnormal aggression.

The electric stimulation of the hypothalamus elicits biting attacks on conspecifics in a variety of species as shown first by Hess (1928) in rats. Systematic studies identified the precise anatomical location of the so-called hypothalamic attack area and helped tremendously in deciphering the neural underpinnings of aggression (Kruk, 1991; Siegel et al., 1999). Optogenetic stimulation, which presents a series of advantages over electric stimulations served the same purposes in the last decade (Anderson, 2012; Falkner et al., 2020). Attacks elicited from the hypothalamus have a series of features that resemble aggression observed in abnormal aggression models. Particularly, attacks are not preceded by social signals, are frequently targeted toward vulnerable body parts of opponents, are not dampened by submissive signals, and do not spare any opponent neither females nor juveniles. However, one can argue that all these are due to the direct activation of the circuitry of aggression, which overrules other factors. As such, hypothalamic aggression is a mechanistic model rather than a true abnormal aggression model. Yet by exploiting the versatility of the optogenetic technique one can selectively elicit either enhanced normal aggression, i.e., that lacks the symptoms of abnormal aggression, or the other way round abnormal aggression that is not associated with increased attack counts (Biro et al., 2018). As such, this approach may be uniquely useful in deciphering the circuits specifically underlying deviant aggression.

Given the tight relationship between stress responses and aggression (Haller, 2022) recent findings on the behavior of rats selected for high glucocorticoid stress responses appear especially pertinent for this study. Selected rats showed increased aggression in terms of both decreased attack latencies and increased bite counts (Walker et al., 2017). A subsequent study by an overlapping set of authors revealed that high stress-responsiveness was associated with symptoms of abnormal aggression (Walker and Sandi, 2018). Interestingly, the high stress responsive rats was less affected by peripubertal stress than the wild-type, suggesting that there is a yet unexplored and to a certain extent counterintuitive interaction between inborn stress responsiveness and the behavioral effects of early stressors.

Finally, I briefly describe the model which raised for the first time the possibility of modeling human psychopathologic aggression in laboratory rodents (Haller et al., 2001). The physiological phenomenon modeled was glucocorticoid deficiency, which was observed first in habitually violent offenders (Virkkunen, 1985) and then in kindergarten-age children, where low glucocorticoid levels predicted aggression problems two years in advance (McBurnett et al., 2000). Subsequent research clarified that chronically low constitutive glucocorticoid production and/or habitually low glucocorticoid stress responses are associated with aggression in a series of disorders, e.g., conduct, oppositional-defiant, antisocial personality, and posttraumatic stress disorders, as well as with violent crime (Haller, 2020, pp. 186–201; Haller, 2022). The findings of McBurnett et al. (2000) were also confirmed by subsequent research, which demonstrated that deficits in glucocorticoid production were followed within a few years by psychopathological and/or criminal aggression. Noteworthy, low glucocorticoid background not only characterized certain aggression-related psychopathologies but also the early subjugation and peripubertal stress models (Ferris et al., 2005; Márquez et al., 2013; Papilloud et al., 2018).

The glucocorticoid deficit was attained in rats by the surgical removal of the adrenals to limit endogenous glucocorticoid secretion and the implantation of subcutaneous corticosterone pellets to prevent neural damages induced by the total absence of glucocorticoids (Haller et al., 2001). As a result, rats had low day-time levels of plasma glucocorticoids without being able to develop glucocorticoid stress responses. Note that rats are nocturnal animals, for which their glucocorticoid levels are about 2-3 times smaller during the day than during the night. Glucocorticoid deficiency did not affect the level of aggression (bite counts remained unchanged), but dramatically increased the share of bites directed toward the vulnerable body parts of opponents, reduced the propensity of animals to signal their attack intentions by threats, and induced social deficits indicative of reduced sociability (Haller et al., 2004, 2005). The short-term inhibition of glucocorticoid secretion by metyrapone had effects opposite to those seen after the chronic treatment, i.e., adrenalectomy (Halasz et al., 2008) and the abolishment of the chronic glucocorticoid deficit by daily glucocorticoid injections also abolished the symptoms of abnormal aggression (Haller et al., 2001). Thus, one can hypothesize that the association between chronic glucocorticoid deficits and abnormal aggression was causal. Importantly for the followings, glucocorticoid deficits were associated with a marked autonomic hypoarousal (Haller et al., 2004). Heart rates were less increased by aggression in glucocorticoid-deficient rats than in controls, and more importantly, heart rates returned to control levels while rats were still fighting, whereas in controls, heart rates remained high long after the aggressive encounter terminated. This is in sharp contrast with the autonomic responses observed in rats submitted to the post-weaning social isolation model.

Taken together, these findings show that mimicking physiological conditions associated with aggression-related psychopathologies induced abnormal aggression just as efficiently as the etiological factors of these disorders.

### Strength and Limitations

The main strength of abnormal aggression models is that they appear relevant from a psychopathological perspective. In general, psychopathologies develop in previously healthy individuals under the effects of environmental insults, e.g., etiological factors, which generate a persistent pattern of emotional and behavioral changes that are inappropriate in the context in which they occur. This phenomenon is rather well modeled in the paradigms presented above. A further strength of these models is that, taken together, they cover all or most of the main etiological factors that are believed to lead to aggression-related psychopathologies.

The main limitation is that the correspondence between the models and psychopathologies remains poorly understood and as such is open to debate. At present, three hypotheses seem to emerge as shown in the next sections.

#### The “Overactivation” Hypothesis

According to this approach, the mechanisms of adaptive and abnormal aggression differ in quantitative terms only (Covington et al., 2019). Certain elements of the adaptive aggression circuits may be up- or downregulated, their connectivity may increase or decrease, but the circuits essentially remain the same. In a way, this hypothesis restores the validity of classical aggression models for understanding psychopathological aggression, and renders abnormal aggression models complementary approaches, which may clarify details unaddressed by classical ones. For instance, an abnormal aggression model may show which component of the basic neural mechanisms of aggression changes under the influence of a particular etiological factor. Although conceivable, and may be true for particular aggression models, mechanistic changes observed in many models go far beyond the quantitative changes assumed by this approach as shown below.

#### The Core Pathway Dichotomy Hypothesis

**FIGURE 2:**
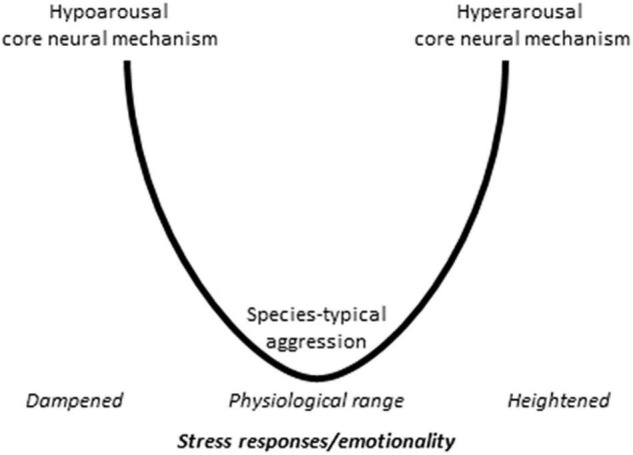
The visualization of the core pathway dichotomy hypothesis. The available data suggest that the mechanisms underlying abnormal aggression follow two basic patterns. The hypoarousal core mechanism may be valid for those abnormal aggression models, which are associated with decreased autonomic/emotional arousal, and consists in the coactivation of the rivalry and predatory aggression neural pathways. The hyperarousal core mechanism can be observed in those models where stress response and emotionality are increased and involves the excessive activation of the rivalry aggression pathways, which is associated with a series anatomical changes in the aggression circuitry.

It was suggested that abnormal aggression models outline two core pathways of abnormal aggression that are differentiated by the emotional component of aggression (Haller, 2013, 2018a,b) (Figure 2).

One core pathway involves the coactivation of the rivalry aggression and predatory aggression circuits. The main axis of the former is the medial prefrontal cortex – medial amygdala – mediobasal hypothalamus – dorsal periaqueductal gray pathway, whereas the main axis of the latter is the orbitofrontal cortex – central amygdala – lateral hypothalamus – ventral periaqueductal gray pathway. This dual activation was observed neither in classical aggression models nor in predatory aggression models but was clearly visible in the glucocorticoid deficit model (Tulogdi et al., 2010, 2015). It was shown subsequently that similar coactivation patterns were operational in a series of other abnormal aggression models, where – like in the glucocorticoid deficit model – aggression was associated with decreased glucocorticoid and autonomic stress responses. Such models were together called the hypoarousal models of abnormal aggression and include mice selected for aggressiveness (short attack latency mice), rats selected for extremes in anxiety, as well as the early subjugation, and the peripubertal stress models (see Haller, 2013, 2018a,b for reviews).

The second core pathway may be associated with less dramatic changes in the controlling mechanisms. This may be the case for abnormal aggression models associated with increased stress responses called hyperarousal models. For example, the first mechanistic study on the post-weaning social isolation model suggested that abnormal aggression and behavioral agitation are associated with the overactivation of the “regular” rivalry aggression circuitry (Toth et al., 2012). As such, this model may conform to the “overactivation” approach presented above. Subsequent research showed, however, that the prefrontal cortex suffered a series of anatomical and functional changes in response to social isolation (Biro et al., 2017). Particularly, the thickness of the infralimbic and prelimbic cortices decreased, and decreases were also observed in the vascularization, glial density and dendritic arborization of these areas. Another study revealed that the methylation status of the brain-derived neurotrophic factor (BDNF) changed in all three the infralimbic cortex, medial amygdala and lateral hypothalamus, the projections from the hippocampus to the prefrontal cortex weakened, and changes were observed in perineuronal nets as well (Mikics et al., 2018). Taken together, these findings suggest that neural changes associated with these models cannot be simply assigned to the overactivation hypothesis. For space limitations, I cannot overview all the hyperarousal models, but it occurs that complex neural changes were observed in all.

Taken together, these findings suggest that (1) abnormal aggression results from complex neuronal changes that are considerably broader that that suggested by the overactivation hypothesis. (2) There are at least two different mechanisms that are associated with, or underlie abnormal aggression. One mechanism may be operational in hypoarousal, whereas the other in hyperarousal models. Tentatively, the two models are laboratory analogs of aggression-related psychopathologies of the proactive and reactive type, e.g., conduct and antisocial personality disorders, psychopathy, as well as violent crime on one side and borderline personality, bipolar and intermittent explosive disorders on the other (Haller, 2020).

#### The Disorder Model Hypothesis

This hypothesis suggest that abnormal aggression models are analogous with specific psychopathologies. For instance, one study proposed that abnormal aggression models should be evaluated based on adapted DSM-5 criteria and suggested that certain abnormal aggression models may be analogous with particular psychopathologies (Haller, 2018c). Another set of studies suggested that attacks on females by peripubertally stressed males may model intimate partner violence and showed that attacked females display behavioral alterations analogous to human victims (Cordero et al., 2012; Poirier et al., 2013). In a similar vein, the premature development of adult aggression in the early subjugation model may be perceived as being analogous to juvenile delinquency, whereas drug-induced abnormal aggression models with human drug-related violence. Such assumptions may occur far-fetched at present but may be workable on the long run.

## Concluding Remarks

The classical models of aggression were of great service when the basic mechanisms of aggression were studied and are still important reference points for the new psychopathology-oriented models. The latter marked a shift from the biological approach to aggression to the psychopathological approach to violence. Many new models were developed over the last two decades, each shedding light to a new aspect of abnormal aggression and violence. The new approach is still suffering from a series of “childhood diseases.” Few models were studied systematically so far, and only a restricted number of findings were crosschecked by independent research groups. In addition, the correspondence between specific models and specific psychopathological states is not entirely clear. I believe, however, that the psychopathology-oriented approach has the potential for understanding the neural mechanisms of abnormal aggression and may significantly contribute to development of novel treatment strategies.

## Author Contributions

The author confirms being the sole contributor of this work and has approved it for publication.

## Conflict of Interest

The author declares that the research was conducted in the absence of any commercial or financial relationships that could be construed as a potential conflict of interest.

## Publisher’s Note

All claims expressed in this article are solely those of the authors and do not necessarily represent those of their affiliated organizations, or those of the publisher, the editors and the reviewers. Any product that may be evaluated in this article, or claim that may be made by its manufacturer, is not guaranteed or endorsed by the publisher.

## References

[B1] Ambar G. Chiavegatto S. (2009). Anabolic-androgenic steroid treatment induces behavioral disinhibition and downregulation of serotonin receptor messenger RNA in the prefrontal cortex and amygdala of male mice. *Genes Brain Behav.* 8 161–173. 10.1111/j.1601-183X.2008.00458.x 19055689

[B2] American Psychiatric Association (2013). *Diagnostic and Statistical Manual of Mental Disorders*, 5th Edn. Washington, DC: American Psychiatric Association.

[B3] Anderson D. J. (2012). Optogenetics, sex, and violence in the brain: implications for psychiatry. *Biol. Psychiatry* 71 1081–1089. 10.1016/j.biopsych.2011.11.012 22209636 PMC3380604

[B4] Bastida C. C. Puga F. Delville Y. (2009). Risk assessment and avoidance in juvenile golden hamsters exposed to repeated stress. *Horm. Behav.* 55 158–162. 10.1016/j.yhbeh.2008.09.009 18948107

[B5] Bastida C. C. Puga F. Gonzalez-Lima F. Jennings K. J. Wommack J. C. Delville Y. (2014). Chronic social stress in puberty alters appetitive male sexual behavior and neural metabolic activity. *Horm Behav.* 66 220–227. 10.1016/j.yhbeh.2014.05.002 24852486 PMC4127097

[B6] Beiderbeck D. I. Reber S. O. Havasi A. Bredewold R. Veenema A. H. Neumann I. D. (2012). High and abnormal forms of aggression in rats with extremes in trait anxiety–involvement of the dopamine system in the nucleus accumbens. *Psychoneuroendocrinology* 37 1969–1980. 10.1016/j.psyneuen.2012.04.011 22608548

[B7] Belfry K. D. Kolla N. J. (2021). Cold-Blooded and on purpose: a review of the biology of proactive aggression. *Brain Sci.* 11:1412. 10.3390/brainsci11111412 34827411 PMC8615983

[B8] Bennett R. M. Buss A. H. Carpenter J. A. (1970). Alcohol and human physical aggression. *Psychiatry Dig.* 31:33. 5454813

[B9] Benus R. F. Bohus B. Koolhaas J. M. van Oortmerssen G. A. (1991). Behavioural differences between artificially selected aggressive and non-aggressive mice: response to apomorphine. *Behav. Brain Res.* 43 203–208. 10.1016/s0166-4328(05)80072-5 1867763

[B10] Biro L. Sipos E. Bruzsik B. Farkas I. Zelena D. Balazsfi D. (2018). Task division within the prefrontal cortex: distinct neuron populations selectively control different aspects of aggressive behavior *via* the hypothalamus. *J. Neurosci.* 38 4065–4075. 10.1523/JNEUROSCI.3234-17.2018 29487128 PMC6596023

[B11] Biro L. Toth M. Sipos E. Bruzsik B. Tulogdi A. Bendahan S. (2017). Structural and functional alterations in the prefrontal cortex after post-weaning social isolation: relationship with species-typical and deviant aggression. *Brain Struct. Funct.* 222 1861–1875. 10.1007/s00429-016-1312-z 27664119

[B12] Blanchard D. C. (2017). Translating dynamic defense patterns from rodents to people. *Neurosci. Biobehav. Rev.* 76(Pt A) 22–28. 10.1016/j.neubiorev.2016.11.001 28434585

[B13] Blanchard R. J. Blanchard D. C. (1989). Attack and defense in rodents as ethoexperimental models for the study of emotion. *Prog. Neuropsychopharmacol. Biol. Psychiatry.* 13(Suppl.) S3–S14. 10.1016/0278-5846(89)90105-x 2694228

[B14] Blanchard R. J. Blanchard D. C. Rodgers J. Weiss S. M. (1990). The characterization and modelling of antipredator defensive behavior. *Neurosci. Biobehav. Rev.* 14 463–472. 10.1016/s0149-7634(05)80069-7 2287483

[B15] Blanchard R. J. Flannelly K. J. Blanchard D. C. (1988). Life-span studies of dominance and aggression in established colonies of laboratory rats. *Physiol. Behav.* 43 1–7. 10.1016/0031-9384(88)90089-3 3413239

[B16] Cairns R. B. MacCombie D. J. Hood K. E. (1983). A developmental-genetic analysis of aggressive behavior in mice: I. Behavioral outcomes. *J. Comp. Psychol.* 97 69–89. 6603330

[B17] Cherek D. R. Steinberg J. L. Kelly T. H. Robinson D. (1987). Effects of d-amphetamine on aggressive responding of normal male subjects. *Psychiatry Res.* 21 257–265. 10.1016/0165-1781(87)90030-8 3628610

[B18] Clotfelter E. D. O’Hare E. P. McNitt M. M. Carpenter R. E. Summers C. H. (2007). Serotonin decreases aggression *via* 5-HT1A receptors in the fighting fish Betta splendens. *Pharmacol. Biochem. Behav.* 87 222–231. 10.1016/j.pbb.2007.04.018 17553555

[B19] Cordero M. I. Ansermet F. Sandi C. (2013). Long-term programming of enhanced aggression by peripuberty stress in female rats. *Psychoneuroendocrinology* 38 2758–2769. 10.1016/j.psyneuen.2013.07.005 23942011

[B20] Cordero M. I. Poirier G. L. Marquez C. Veenit V. Fontana X. Salehi B. (2012). Evidence for biological roots in the transgenerational transmission of intimate partner violence. *Transl. Psychiatry.* 2:e106. 10.1038/tp.2012.32 22832906 PMC3337076

[B21] Covington H. E. III Newman E. L. Leonard M. Z. Miczek K. A. (2019). Translational models of adaptive and excessive fighting: an emerging role for neural circuits in pathological aggression. *F1000Res* 8:F1000 FacultyRev-963. 10.12688/f1000research.18883.1 31281636 PMC6593325

[B22] Craig W. (1917). Appetites and aversions as constituents of instincts. *Proc. Natl. Acad. Sci. U.S.A.* 3 685–688. 10.1073/pnas.3.12.685 16586767 PMC1091358

[B23] Cunningham R. L. McGinnis M. Y. (2008). Prepubertal social subjugation and anabolic androgenic steroid-induced aggression in male rats. *J. Neuroendocrinol.* 20 997–1005. 10.1111/j.1365-2826.2008.01756.x 18510706

[B24] da Cunha-Bang S. Knudsen G. M. (2021). The modulatory role of serotonin on human impulsive aggression. *Biol. Psychiatry* 90 447–457. 10.1016/j.biopsych.2021.05.016 34266672

[B25] Deckel A. W. (1996). Behavioral changes in *Anolis carolinensis* following injection with fluoxetine. *Behav. Brain Res.* 78 175–182. 10.1016/0166-4328(95)00246-4 8864049

[B26] Delville Y. Melloni R. H. Jr. Ferris C. F. (1998). Behavioral and neurobiological consequences of social subjugation during puberty in golden hamsters. *J. Neurosci.* 18 2667–2672. 10.1523/JNEUROSCI.18-07-02667.1998 9502824 PMC6793084

[B27] Dennis R. L. Lay D. C. Jr. Cheng H. W. (2013). Effects of early serotonin programming on behavior and central monoamine concentrations in an avian model. *Behav. Brain Res.* 253 290–296. 10.1016/j.bbr.2013.07.043 23912030

[B28] Edsinger E. Dölen G. A. (2018). Conserved role for serotonergic neurotransmission in mediating social behavior in octopus. *Curr. Biol.* 28 3136–3142.e4. 10.1016/j.cub.2018.07.061 30245101 PMC13460050

[B29] Estrada-Martínez L. M. Padilla M. B. Caldwell C. H. Schulz A. J. (2011). Examining the influence of family environments on youth violence: a comparison of Mexican, Puerto Rican, Cuban, non-Latino Black, and non-Latino White adolescents. *J. Youth Adolesc.* 40 1039–1051. 10.1007/s10964-010-9624-4 21188487

[B30] Falkner A. L. Wei D. Song A. Watsek L. W. Chen I. Chen P. (2020). Hierarchical representations of aggression in a hypothalamic-midbrain circuit. *Neuron* 106 637–648.e6. 10.1016/j.neuron.2020.02.014 32164875 PMC7571490

[B31] Ferris C. F. Messenger T. Sullivan R. (2005). Behavioral and neuroendocrine consequences of social subjugation across adolescence and adulthood. *Front. Zool.* 2:7. 10.1186/1742-9994-2-7 15847686 PMC1090605

[B32] Fitzpatrick S. M. Wellington W. G. (1983). Insect territoriality. *Can. J. Zool.* 61 471–486. 10.1139/z83-064

[B33] Freedman D. Hemenway D. (2000). Precursors of lethal violence: a death row sample. *Soc. Sci. Med.* 50 1757–1770. 10.1016/s0277-9536(99)00417-7 10798330

[B34] Gandelman R. (1972). Mice: postpartum aggression elicited by the presence of an intruder. *Horm. Behav.* 3 23–28. 10.1016/0018-506x(72)90003-7 4681734

[B35] Glowacki L. McDermott R. (2022). Key individuals catalyse intergroup violence. *Philos. Trans. R. Soc. Lond. B Biol. Sci.* 377:20210141. 10.1098/rstb.2021.0141 35369758 PMC8977664

[B36] Gobrogge K. L. Liu Y. Young L. J. Wang Z. (2009). Anterior hypothalamic vasopressin regulates pair-bonding and drug-induced aggression in a monogamous rodent. *Proc. Natl. Acad. Sci. U.S.A.* 106 19144–19149. 10.1073/pnas.0908620106 19858480 PMC2776424

[B37] Gostisha A. J. Vitacco M. J. Dismukes A. R. Brieman C. Merz J. Shirtcliff E. A. (2014). Beyond physiological hypoarousal: the role of life stress and callous-unemotional traits in incarcerated adolescent males. *Horm. Behav.* 65 469–479. 10.1016/j.yhbeh.2014.03.016 24726789 PMC4580972

[B38] Halasz J. Toth M. Mikics E. Hrabovszky E. Barsy B. Barsvari B. (2008). The effect of neurokinin1 receptor blockade on territorial aggression and in a model of violent aggression. *Biol. Psychiatry* 63 271–278. 10.1016/j.biopsych.2007.04.022 17678879

[B39] Haller J. (2013). The neurobiology of abnormal manifestations of aggression–a review of hypothalamic mechanisms in cats, rodents, and humans. *Brain Res. Bull.* 93 97–109. 10.1016/j.brainresbull.2012.10.003 23085544

[B40] Haller J. (2017). Studies into abnormal aggression in humans and rodents: methodological and translational aspects. *Neurosci. Biobehav. Rev.* 76(Pt A) 77–86. 10.1016/j.neubiorev.2017.02.022 28434590

[B41] Haller J. (2018a). The role of the lateral hypothalamus in violent intraspecific aggression-the glucocorticoid deficit hypothesis. *Front. Syst. Neurosci.* 12:26. 10.3389/fnsys.2018.00026 29937719 PMC6002688

[B42] Haller J. (2018b). The role of central and medial amygdala in normal and abnormal aggression: a review of classical approaches. *Neurosci. Biobehav. Rev.* 85 34–43. 10.1016/j.neubiorev.2017.09.017 28918358

[B43] Haller J. (2018c). Preclinical models of conduct disorder - principles and pharmacologic perspectives. *Neurosci. Biobehav. Rev.* 91 112–120. 10.1016/j.neubiorev.2016.05.032 27238913

[B44] Haller J. (2020). *Neurobiopsychosocial Perspectives on Aggression and Violence. From Biology to Law Enforcement.* Cham: Springer.

[B45] Haller J. (2022). Glucocorticoids and aggression: a tripartite interaction. *Curr. Top. Behav. Neurosci.* 54 209–243. 10.1007/7854_2022_307 35362871

[B46] Haller J. Kruk M. R. (2006). Normal and abnormal aggression: human disorders and novel laboratory models. *Neurosci. Biobehav. Rev.* 30 292–303. 10.1016/j.neubiorev.2005.01.005 16483889

[B47] Haller J. Halász J. Mikics E. Kruk M. R. (2004). Chronic glucocorticoid deficiency-induced abnormal aggression, autonomic hypoarousal, and social deficit in rats. *J. Neuroendocrinol.* 16 550–557. 10.1111/j.1365-2826.2004.01201.x 15189330

[B48] Haller J. Tóth M. Halász J. (2005). The activation of raphe serotonergic neurons in normal and hypoarousal-driven aggression: a double labeling study in rats. *Behav. Brain Res.* 161 88–94. 10.1016/j.bbr.2005.01.006 15904714

[B49] Haller J. Tóth M. Halasz J. De Boer S. F. (2006). Patterns of violent aggression-induced brain c-fos expression in male mice selected for aggressiveness. *Physiol. Behav.* 88 173–182. 10.1016/j.physbeh.2006.03.030 16687160

[B50] Haller J. van de Schraaf J. Kruk M. R. (2001). Deviant forms of aggression in glucocorticoid hyporeactive rats: a model for ‘pathological’ aggression? *J. Neuroendocrinol.* 13 102–107. 10.1046/j.1365-2826.2001.00600.x 11123520

[B51] Harlow H. F. (1965). Total social isolation: effects on macaque monkey behavior. *Science* 148:666. 10.1126/science.148.3670.666-a 17801949

[B52] Herrenkohl T. L. Hawkins J. D. Chung I. Hill K. G. Battin-Pearson S. (2001). “School and community risk factors and interventions,” in *Child delinquents: Development, Intervention, and Service Needs*, eds Loeber R. Farrington D. P. (Thousand Oaks, CA: Sage), 211–246.

[B53] Hess W. R. (1928). Stammganglien-reizversuche. *Berichte Gesamt. Physiol.* 42 554–555.

[B54] Hicks B. M. Carlson M. D. Blonigen D. M. Patrick C. J. Iacono W. G. Mgue M. (2012). Psychopathic personality traits and environmental contexts: differential correlates, gender differences, and genetic mediation. *Personal. Disord.* 3 209–227. 10.1037/a0025084 22452762 PMC3387315

[B55] Huffard C. L. Caldwell R. L. Boneka F. (2010). Male-male and male-female aggression may influence mating associations in wild octopuses (*Abdopus aculeatus*). *J. Comp. Psychol.* 124 38–46. 10.1037/a0017230 20175595

[B56] Jalabert C. Munley K. Demas G. Soma K. (2018). “Aggressive behavior,” in *Encyclopedia of Reproduction*, Vol. 1 ed. Anderson C. (Hoboken, NJ: Wiley Online Library), 242–247.

[B57] Kaiser S. Harderthauer S. Sachser N. Hennessy M. B. (2007). Social housing conditions around puberty determine later changes in plasma cortisol levels and behavior. *Physiol. Behav.* 90 405–411. 10.1016/j.physbeh.2006.10.002 17196999

[B58] Koolhaas J. M. Coppens C. M. de Boer S. F. Buwalda B. Meerlo P. Timmermans P. J. (2013). The resident-intruder paradigm: a standardized test for aggression, violence and social stress. *J. Vis. Exp.* 77:e4367. 10.3791/4367 23852258 PMC3731199

[B59] Kruk M. R. (1991). Ethology and pharmacology of hypothalamic aggression in the rat. *Neurosci. Biobehav. Rev.* 15 527–538. 10.1016/s0149-7634(05)80144-7 1792015

[B60] Lagerspetz K. M. Lagerspetz K. Y. (1971). Changes in the aggressiveness of mice resulting from selective breeding, learning and social isolation. *Scand. J. Psychol.* 12 241–248. 10.1111/j.1467-9450.1971.tb00627.x 5170173

[B61] Lee T. J. Zanello A. F. Morrison T. R. Ricci L. A. Melloni R. H. Jr. (2021). Valproate selectively suppresses adolescent anabolic/androgenic steroid-induced aggressive behavior: implications for a role of hypothalamic γ-aminobutyric acid neural signaling. *Behav. Pharmacol.* 32 295–307. 10.1097/FBP.0000000000000616 33595952

[B62] Licata A. Taylor S. Berman M. Cranston J. (1993). Effects of cocaine on human aggression. *Pharmacol. Biochem. Behav.* 45 549–552. 10.1016/0091-3057(93)90504-m 8332615

[B63] Liebsch G. Montkowski A. Holsboer F. Landgraf R. (1998). Behavioural profiles of two Wistar rat lines selectively bred for high or low anxiety-related behaviour. *Behav. Brain Res.* 94 301–310. 10.1016/s0166-4328(97)00198-8 9722280

[B64] Mamiya P. C. Matray-Devoti J. Fisher H. Wagner G. C. (2017). Mice increased target biting behaviors 24h after co-administration of alcohol and fluoxetine. *Brain Res.* 1662 110–115. 10.1016/j.brainres.2017.02.007 28193480

[B65] Márquez C. Poirier G. L. Cordero M. I. Larsen M. H. Groner A. Marquis J. (2013). Peripuberty stress leads to abnormal aggression, altered amygdala and orbitofrontal reactivity and increased prefrontal MAOA gene expression. *Transl. Psychiatry* 3:e216. 10.1038/tp.2012.144 23321813 PMC3566724

[B66] McBurnett K. Lahey B. B. Rathouz P. J. Loeber R. (2000). Low salivary cortisol and persistent aggression in boys referred for disruptive behavior. *Arch. Gen. Psychiatry* 57 38–43. 10.1001/archpsyc.57.1.38 10632231

[B67] McGuire J. (2008). A review of effective interventions for reducing aggression and violence. *Philos. Trans. R. Soc. Lond. B Biol. Sci.* 363 2577–2597. 10.1098/rstb.2008.0035 18467276 PMC2606715

[B68] McLaughlin K. A. Sheridan M. A. Nelson C. A. (2017). Neglect as a violation of species-expectant experience: neurodevelopmental consequences. *Biol. Psychiatry.* 82 462–471. 10.1016/j.biopsych.2017.02.1096 28392082 PMC5572554

[B69] Meier B. P. Wilkowski B. M. (2013). Reducing the tendency to aggress: insights from social and personality psychology. *Soc. Personal. Psychol. Compass* 7 343–354. 10.1111/spc3.12029

[B70] Melloni R. H. Jr. Ferris C. F. (1996). Adolescent anabolic steroid use and aggressive behavior in golden hamsters. *Ann. N. Y. Acad. Sci.* 794 372–375. 10.1111/j.1749-6632.1996.tb32546.x 8853620

[B71] Meyer J. M. Cummings M. A. Proctor G. Stahl S. M. (2016). Psychopharmacology of persistent violence and aggression. *Psychiatr. Clin. North Am.* 39 541–556. 10.1016/j.psc.2016.07.012 27836150

[B72] Miczek K. A. de Boer S. F. Haller J. (2013). Excessive aggression as model of violence: a critical evaluation of current preclinical methods. *Psychopharmacology (Berl.)* 226 445–458. 10.1007/s00213-013-3008-x 23430160 PMC3595336

[B73] Miczek K. A. Weerts E. M. Tornatzky W. DeBold J. F. Vatne T. M. (1992). Alcohol and “bursts” of aggressive behavior: ethological analysis of individual differences in rats. *Psychopharmacology (Berl.)* 107 551–563. 10.1007/BF02245270 1603899

[B74] Mikics É Guirado R. Umemori J. Tóth M. Biró L. Miskolczi C. (2018). Social learning requires plasticity enhanced by fluoxetine through prefrontal Bdnf-TrkB signaling to limit aggression induced by post-weaning social isolation. *Neuropsychopharmacology* 43 235–245. 10.1038/npp.2017.142 28685757 PMC5635971

[B75] Mikics E. Barsy B. Haller J. (2007). The effect glucocorticoids on aggressiveness in established colonies of rats. *Psychoneuroendocrinology* 32 160–170. 10.1016/j.psyneuen.2006.12.002 17275197

[B76] Millard A. Gentsch C. (2006). Competition for sucrose pellets in tetrads of male wistar, fischer or sprague-dawley rats: is intra-group ranking reflected in the level of anxiety? *Behav. Brain Res.* 168 243–254. 10.1016/j.bbr.2005.11.012 16360888

[B77] Morrison T. R. Ricci L. A. Melloni R. H. Jr. (2014). γ-Aminobutyric acid neural signaling in the lateroanterior hypothalamus modulates aggressive behavior in adolescent anabolic/androgenic steroid-treated hamsters. *Behav. Pharmacol.* 25 673–683. 10.1097/FBP.0000000000000083 25171080 PMC4331350

[B78] Mosienko V. Bert B. Beis D. Matthes S. Fink H. Bader M. (2012). Exaggerated aggression and decreased anxiety in mice deficient in brain serotonin. *Transl. Psychiatry.* 2:e122. 10.1038/tp.2012.44 22832966 PMC3365263

[B79] Natarajan D. Caramaschi D. (2010). Animal violence demystified. *Front. Behav. Neurosci.* 4:9. 10.3389/fnbeh.2010.00009 20407576 PMC2854525

[B80] Natarajan D. de Vries H. de Boer S. F. Koolhaas J. M. (2009). Violent phenotype in SAL mice is inflexible and fixed in adulthood. *Aggress. Behav.* 35 430–436. 10.1002/ab.20312 19533684

[B81] Neumann I. D. Veenema A. H. Beiderbeck D. I. (2010). Aggression and anxiety: social context and neurobiological links. *Front. Behav. Neurosci.* 4:12. 10.3389/fnbeh.2010.00012 20407578 PMC2854527

[B82] New A. S. Hazlett E. A. Buchsbaum M. S. Goodman M. Mitelman S. A. Newmark R. (2007). Amygdala-prefrontal disconnection in borderline personality disorder. *Neuropsychopharmacology* 32 1629–1640. 10.1038/sj.npp.1301283 17203018

[B83] Newman E. L. Terunuma M. Wang T. L. Hewage N. Bicakci M. B. Moss S. J. (2018). Role for prefrontal Cortical NMDA receptors in murine alcohol-heightened aggression. *Neuropsychopharmacology* 43 1224–1234. 10.1038/npp.2017.253 29052618 PMC5916347

[B84] Oliveira V. E. M. Lukas M. Wolf H. N. Durante E. Lorenz A. Mayer A. L. (2021). Oxytocin and vasopressin within the ventral and dorsal lateral septum modulate aggression in female rats. *Nat. Commun.* 12:2900. 10.1038/s41467-021-23064-5 34006875 PMC8131389

[B85] Oliveira V. E. M. Neumann I. D. de Jong T. R. (2019). Post-weaning social isolation exacerbates aggression in both sexes and affects the vasopressin and oxytocin system in a sex-specific manner. *Neuropharmacology* 156:107504. 10.1016/j.neuropharm.2019.01.019 30664846

[B86] Papilloud A. Guillot de Suduiraut I. Zanoletti O. Grosse J. Sandi C. (2018). Peripubertal stress increases play fighting at adolescence and modulates nucleus accumbens CB1 receptor expression and mitochondrial function in the amygdala. *Transl. Psychiatry* 8:156. 10.1038/s41398-018-0215-6 30111823 PMC6093900

[B87] Parmigiani S. Brain P. F. Mainardi D. Brunoni V. (1988). Different patterns of biting attack employed by lactating female mice (*Mus domesticus*) in encounters with male and female conspecific intruders. *J. Comp. Psychol.* 102 287–293. 10.1037/0735-7036.102.3.287 3180736

[B88] Plyusnina I. Oskina I. (1997). Behavioral and adrenocortical responses to open-field test in rats selected for reduced aggressiveness toward humans. *Physiol. Behav.* 61 381–385. 10.1016/s0031-9384(96)00445-3 9089756

[B89] Poirier G. L. Cordero M. I. Sandi C. (2013). Female vulnerability to the development of depression-like behavior in a rat model of intimate partner violence is related to anxious temperament, coping responses, and amygdala vasopressin receptor 1a expression. *Front. Behav. Neurosci.* 7:35. 10.3389/fnbeh.2013.00035 23641204 PMC3640184

[B90] Potegal M. Einon D. (1989). Aggressive behaviors in adult rats deprived of playfighting experience as juveniles. *Dev. Psychobiol.* 22 159–172. 10.1002/dev.420220206 2925003

[B91] Powell D. A. Francis J. Braman M. J. Schneiderman N. (1969). Frequency of attack in shock-elicited aggression as a function of the performance of individual rats. *J. Exp. Anal. Behav.* 12 817–823. 10.1901/jeab.1969.12-817 5391063 PMC1338685

[B92] Raine A. Yang Y. (2006). Neural foundations to moral reasoning and antisocial behavior. *Soc. Cogn. Affect. Neurosci.* 1 203–213. 10.1093/scan/nsl033 18985107 PMC2555414

[B93] Reinhard J. Rowell D. M. (2005). Social behaviour in an Australian velvet worm, *Euperipatoides rowelli* (Onychophora : Peripatopsidae). *J. Zool.* 267 1–7. 10.1017/S0952836905007090

[B94] Ricci L. A. Grimes J. M. Knyshevski I. Melloni R. H. (2005). Repeated cocaine exposure during adolescence alters glutamic acid decarboxylase-65 (GAD65) immunoreactivity in hamster brain: correlation with offensive aggression. *Brain Res.* 1035 131–138. 10.1016/j.brainres.2004.11.049 15722053

[B95] Rizzi M. Gambini O. Marras C. E. (2021). Posterior hypothalamus as a target in the treatment of aggression: from lesioning to deep brain stimulation. *Handb. Clin. Neurol.* 182 95–106. 10.1016/B978-0-12-819973-2.00007-1 34266615

[B96] Rosenström T. Ystrom E. Torvik F. A. Czajkowski N. O. Gillespie N. A. Aggen S. H. (2017). Genetic and environmental Structure of DSM-IV criteria for antisocial personality disorder: a twin study. *Behav. Genet.* 47 265–277. 10.1007/s10519-016-9833-z 28108863 PMC5404958

[B97] Salatino-Oliveira A. Murray J. Kieling C. Genro J. P. Polanczyk G. Anselmi L. (2016). COMT and prenatal maternal smoking in associations with conduct problems and crime: the Pelotas 1993 birth cohort study. *Sci. Rep.* 6:29900. 10.1038/srep29900 27426045 PMC4947962

[B98] Sandi C. Haller J. (2015). Stress and the social brain: behavioural effects and neurobiological mechanisms. *Nat. Rev. Neurosci.* 16 290–304. 10.1038/nrn3918 25891510

[B99] Savolainen J. Eisman A. Mason W. A. Schwartz J. A. Miettunen J. Järvelin M. R. (2018). Socioeconomic disadvantage and psychological deficits: pathways from early cumulative risk to late-adolescent criminal conviction. *J. Adolesc.* 65 16–24. 10.1016/j.adolescence.2018.02.010 29522913 PMC6143898

[B100] Schweinfurth M. K. (2020). The social life of Norway rats (*Rattus norvegicus*). *eLife* 9:e54020. 10.7554/eLife.54020 32271713 PMC7145424

[B101] Siegel A. Douard J. (2011). Who’s flying the plane: serotonin levels, aggression and free will. *Int. J. Law Psychiatry* 34 20–29. 10.1016/j.ijlp.2010.11.004 21112635 PMC3034832

[B102] Siegel A. Roeling T. A. Gregg T. R. Kruk M. R. (1999). Neuropharmacology of brain-stimulation-evoked aggression. *Neurosci. Biobehav. Rev.* 23 359–389. 10.1016/s0149-7634(98)00040-2 9989425

[B103] Su T. P. Pagliaro M. Schmidt P. J. Pickar D. Wolkowitz O. Rubinow D. R. (1993). Neuropsychiatric effects of anabolic steroids in male normal volunteers. *JAMA* 269 2760–2764. 8492402

[B104] Ten Eyck G. R. (2008). Serotonin modulates vocalizations and territorial behavior in an amphibian. *Behav. Brain Res.* 193 144–147. 10.1016/j.bbr.2008.05.001 18554729

[B105] Thurmond J. B. (1975). Technique for producing and measuring territorial aggression using laboratory mice. *Physiol. Behav.* 14 879–881. 10.1016/0031-9384(75)90086-4 1237905

[B106] Tóth M. Halász J. Mikics E. Barsy B. Haller J. (2008). Early social deprivation induces disturbed social communication and violent aggression in adulthood. *Behav. Neurosci.* 122 849–854. 10.1037/0735-7044.122.4.849 18729638

[B107] Toth M. Mikics E. Tulogdi A. Aliczki M. Haller J. (2011). Post-weaning social isolation induces abnormal forms of aggression in conjunction with increased glucocorticoid and autonomic stress responses. *Horm. Behav.* 60 28–36. 10.1016/j.yhbeh.2011.02.003 21316368

[B108] Toth M. Tulogdi A. Biro L. Soros P. Mikics E. Haller J. (2012). The neural background of hyper-emotional aggression induced by post-weaning social isolation. *Behav. Brain Res.* 233 120–129. 10.1016/j.bbr.2012.04.025 22548916

[B109] Tulogdi A. Biro L. Barsvari B. Stankovic M. Haller J. Toth M. (2015). Neural mechanisms of predatory aggression in rats-implications for abnormal intraspecific aggression. *Behav. Brain Res.* 283 108–115. 10.1016/j.bbr.2015.01.030 25637071

[B110] Tulogdi A. Tóth M. Barsvári B. Biró L. Mikics E. Haller J. (2014). Effects of resocialization on post-weaning social isolation-induced abnormal aggression and social deficits in rats. *Dev. Psychobiol.* 56 49–57. 10.1002/dev.21090 23168609

[B111] Tulogdi A. Toth M. Halasz J. Mikics E. Fuzesi T. Haller J. (2010). Brain mechanisms involved in predatory aggression are activated in a laboratory model of violent intra-specific aggression. *Eur. J. Neurosci.* 32 1744–1753. 10.1111/j.1460-9568.2010.07429.x 21039962

[B112] van Oortmerssen G. A. Bakker T. C. (1981). Artificial selection for short and long attack latencies in wild *Mus musculus* domesticus. *Behav. Genet.* 11 115–126. 10.1007/BF01065622 7196726

[B113] Viding E. Price T. S. Jaffee S. R. Trzaskowski M. Davis O. S. Meaburn E. L. (2013). Genetics of callous-unemotional behavior in children. PLoS One. 2013 Jul 9;8(7):e65789. Erratum in. *PLoS One* 8:e65789. 10.1371/annotation/0b16418f-ceb5-41b2-be2a-a20f0c56f9a6 PMC3706442 23874384

[B114] Virkkunen M. (1985). Urinary free cortisol secretion in habitually violent offenders. *Acta Psychiatr. Scand.* 72 40–44. 10.1111/j.1600-0447.1985.tb02568.x 2994368

[B115] Walker S. E. Sandi C. (2018). Long-term programing of psychopathology-like behaviors in male rats by peripubertal stress depends on individual’s glucocorticoid responsiveness to stress. *Stress* 21 433–442. 10.1080/10253890.2018.1435639 29415604

[B116] Walker S. E. Wood T. C. Cash D. Mesquita M. Williams S. C. R. Sandi C. (2018). Alterations in brain microstructure in rats that develop abnormal aggression following peripubertal stress. *Eur. J. Neurosci.* 48 1818–1832. 10.1111/ejn.14061 29961949

[B117] Walker S. E. Zanoletti O. Guillot de Suduiraut I. Sandi C. (2017). Constitutive differences in glucocorticoid responsiveness to stress are related to variation in aggression and anxiety-related behaviors. *Psychoneuroendocrinology* 84 1–10. 10.1016/j.psyneuen.2017.06.011 28647673

[B118] Wommack J. C. Delville Y. (2003). Repeated social stress and the development of agonistic behavior: individual differences in coping responses in male golden hamsters. *Physiol. Behav.* 80 303–308. 10.1016/j.physbeh.2003.08.002 14637229

[B119] World Health Organization [WHO] (2015). *Regional Office for Europe, European Detailed Mortality Database.* Available online at: https://www.euro.who.int/en/health-topics/disease-prevention/violence-and-injuries (accessed April 04, 2022).

[B120] World Health Organization [WHO] (2022). *Global Health Observatory Depository.* Available online at: http://apps.who.int/gho/data/node.main.CODWORLD?lang=en (accessed April 04, 2022).

[B121] Wright K. A. Turanovic J. J. O’Neal E. N. Morse S. J. Booth E. T. (2016). The cycle of violence revisited: childhood victimization, resilience, and future violence. *J. Interpers. Violence* 34 1261–1286. 10.1177/0886260516651090 27229918

[B122] Young S. E. Friedman N. P. Miyake A. Willcutt E. G. Corley R. P. Haberstick B. C. (2009). Behavioral disinhibition: liability for externalizing spectrum disorders and its genetic and environmental relation to response inhibition across adolescence. *J. Abnorm. Psychol.* 118 117–130. 10.1037/a0014657 19222319 PMC2775710

